# Emotional Intent Modulates The Neural Substrates Of Creativity: An fMRI Study of Emotionally Targeted Improvisation in Jazz Musicians

**DOI:** 10.1038/srep18460

**Published:** 2016-01-04

**Authors:** Malinda J. McPherson, Frederick S. Barrett, Monica Lopez-Gonzalez, Patpong Jiradejvong, Charles J. Limb

**Affiliations:** 1 Department of Otolaryngology-Head and Neck Surgery, Johns Hopkins School of Medicine, Baltimore, Maryland, 21205, USA; 2 Department of Psychiatry and Behavioral Sciences, Johns Hopkins School of Medicine, Baltimore, Maryland, 21205, USA; 3 Peabody Conservatory of The Johns Hopkins University, Baltimore, Maryland, 21202, USA; 4 Department of Otolaryngology-Head and Neck Surgery, University of California San Francisco, San Francisco, California, 94143, USA

## Abstract

Emotion is a primary motivator for creative behaviors, yet the interaction between the neural systems involved in creativity and those involved in emotion has not been studied. In the current study, we addressed this gap by using fMRI to examine piano improvisation in response to emotional cues. We showed twelve professional jazz pianists photographs of an actress representing a positive, negative or ambiguous emotion. Using a non-ferromagnetic thirty-five key keyboard, the pianists improvised music that they felt represented the emotion expressed in the photographs. Here we show that activity in prefrontal and other brain networks involved in creativity is highly modulated by emotional context. Furthermore, emotional intent directly modulated functional connectivity of limbic and paralimbic areas such as the amygdala and insula. These findings suggest that emotion and creativity are tightly linked, and that the neural mechanisms underlying creativity may depend on emotional state.

The ability of art to serve as a means of emotional self-expression and emotional communication has arguably been one of the fundamental reasons for the omnipresence of art throughout all cultures in human history^1^,^2^. Creative mediums such as painting, poetry, dance, film and music evoke intense emotions for both artists and audiences alike, allowing humans to experience and share a wide range of emotional responses within a secure framework^3^. Emotion often serves as a catalyst for creative expression^4^,^5^, and therefore it is crucial to understand how emotion impacts the neural mechanisms that give rise to creativity, and also to understand how creative artistic expression can modulate the neural systems responsible for processing emotions. Here we examined the effects of emotional intent on the neural systems involved in the creation of emotional music compositions using functional magnetic resonance imaging (fMRI). We instructed professional jazz pianists to improvise short pieces in response to visually presented emotional cues with positive, ambiguous, and negative valences (Fig. 1b). Using this paradigm, we examined whether emotional intent influences the neural substrates of creativity.

To our knowledge, this experiment represents the first effort in neuroscience to address the relationship between musical creativity and emotional expression. The majority of the literature on both emotion and music (and emotion in other domains) has examined the perception of emotions rather than the expression of emotions. This is a major deficit in the literature, as a comprehensive model of human emotion processing must include both perception and expression (production) of emotional states, two distinct yet intrinsically linked human experiences. Since the creative expression of emotions through music and other artistic mediums is often used to regulate, project, and alter emotional states^6^, art may be arguably the best means by which to first approach the complex relationships between creativity and emotion, and how they interact neurologically.

Creativity can be broadly defined as the ability to produce something that is both novel and valuable for a given context^7^. Part of the difficulty in studying and modeling creativity is that the nature of creative pursuits in the arts is not bound by notions of correct or incorrect, right or wrong, or even better or worse; indeed violations of expectation are a key criteria used to determine whether something is considered creative^8^ and art critics throughout history have harshly criticized works of art that eventually came to be regarded as masterpieces^9^. In music, violations of expectation (surprises) are often a critical feature of its emotional content^10^,^11^,^12^. Improvisation is a form of immediate creativity in which artists can spontaneously generate novel material, and is a defining feature of jazz music. Jazz musicians are highly trained experts in improvisation, and they are able to reliably perform emotionally targeted improvisations in a laboratory setting^13^. Jazz is a diverse and unrestrictive form of music, and jazz improvisation enables musicians to flexibly incorporate many musical features in order to express an emotion^14^,^15^. Therefore, musical improvisation offers a unique opportunity to systematically examine the effects of emotion on creativity (and creativity on emotion) in an ecologically valid setting.

A growing number of researchers have studied neural activation during creative tasks in both musical and nonmusical domains, however none have directly addressed the role of expressing emotion through naturalistic improvisation^16^,^17^,^18^,^19^,^20^,^21^,^22^,^23^. Multiple studies have reported improvisation-related activations in the premotor cortex, supplementary motor area (SMA), perisylvian language areas, and the medial prefrontal cortex. Some of these studies have reported deactivations during improvisation in the dorsolateral prefrontal cortex (DLPFC) and parietal areas, including the angular gyrus (AG) and precuneus. Deactivation in the DLPFC may indicate that creativity can induce a state of total immersion often referred to as a “flow state”^24^. More widespread DLPFC deactivation, referred to as hypofrontality, may be indicative of deeper flow states^25^. Creativity is not a single unified set of mental processes or abilities. While some types of creativity may require intense concentration and thought, other forms of creativity, such as jazz improvisation, may be predicated on “letting go”^8^. Hypofrontality may represent a neural signature of these types of creative experiences^25^. A recent study by Pinho and colleagues^23^ used an emotional constraint (happy or fearful) vs. a tonal constraint (six tonal or atonal pitches), and confirmed findings that DLPFC activity is lower during free improvisation when compared to tightly constrained improvisation. While emotion was a factor in their study, their goal was to examine the differences between a more loosely constrained, emotional improvisation task, and a more constrained improvisation task, where the pitches were pre-determined. This differs from the current study’s goal of examining differences between free improvisations with different emotional targets.

Similarities between previous studies of creativity suggest that there is a consistent functional network of brain areas responsible for artistic creativity. Yet the existence of this functional network does not reveal how it can produce such a diverse range of creative behaviors that are fundamentally unpredictable. Consequently, identification of the factors that modulate this network is critically important in understanding the capacity of the brain to generate the wide range of ideas that are characteristic of human creativity. In light of the central role of emotion in creative pursuits, we sought to identify whether or not emotional intent modulates the neural systems involved in creativity, possibly lending support to a dynamic neural model of creativity that is malleable in response to numerous external influences.

We hypothesized that patterns of activation of the distributed network of brain regions involved in musical improvisation (compared to a chromatic scale performance control condition) as well as functional connectivity between this network and brain areas that support emotion processing would be modulated by emotional intent. As flow states are generally associated with pleasurable experiences^24^, and performing both happy and sad music can be intensely pleasurable^26^,^27^, we predicted that there would be stronger engagement of reward and flow state areas of the brain during both positive and negative improvisation when compared to ambiguous improvisation. We addressed these hypotheses using an ecologically valid model of spontaneous musical performance in expert jazz pianists improvising in response to emotional targets during fMRI brain scanning.

## Results

### Music Performance Analysis

Analysis of musical mode showed that 31.25% of the negative improvisations were in a major key compared with 79.17% of the positive improvisations and 45.83% of ambiguous improvisations (Fig. 2a).

A main effect of emotion was found for note minima [F(1, 3) = 4.04, *p* < 0.05] and note maxima [F(1, 3) = 8.49, *p* < 0.001]. This was primarily due to differences between negative and positive improvisations (significant for both maxima and minima (*p* < 0.001), as ambiguous improvisations were not statistically different from positive or negative improvisation trials (Fig. 2b).

We observed statistical differences (*p* < 0.05) between corresponding bins of the duration distributions for negative, positive and ambiguous improvisations in all but Bin 3 (¼ s; no significant difference between ambiguous and positive, *p <* 0.05), Bin 4 and Bin 6 (approximately 7/20 s and 14/20 s respectively, with no significant differences between ambiguous and negative, *p <* 0.05) (Fig. 2c).

A main effect of emotion was found for note densities between improvisation conditions [*F*(1, 3) = 31.88, *p* < 0.001]. There was a significant difference between the note densities of positive and negative improvisation conditions and ambiguous and positive improvisation conditions (*p* < 0.001). Higher note densities were used to express positive emotions, and lower note densities were used to express negative and ambiguous emotions (mean note densities: negative = 2.09, ambiguous = 2.56, positive = 3.61). There was no main effect of emotion for note density during chromatic scale trials [*F*(1, 3) = 0.36, *p* = 0.68] (Fig. 2d).

### Functional Neuroimaging: Contrast analyses

Emotional intent modulates activity in brain regions associated with improvisation. To examine brain areas involved in improvisation within each emotion condition we completed within-emotion contrasts (ex. [PosImprov > PosChrom]). We observed activations in left Broca’s area (BA 45) in all emotion conditions and SMA (BA 6) for ambiguous and negative improvisation, as well as deactivation in bilateral AG (BA 40), middle precuneus (BA 7), medial and lateral frontopolar prefrontal cortex (BA 10), and DLPFC (BA 9) for all emotion conditions. There were deactivations in the left hippocampus during positive contrasts, right hippocampus during ambiguous, and bilateral hippocampus during negative contrasts. Additionally, we saw bilateral middle cingulate (BA 31) deactivations for all emotions. Though these activations and deactivations were present among all emotions (SMA notwithstanding), the extent of the voxel clusters varied widely between each emotion. Most notably, positive improvisation showed much more widespread deactivation in the DLPFC, AG, and precuneus than negative and ambiguous improvisation (Fig. 3, Table 1).

These within-emotion contrasts also revealed clusters that were emotion-specific (i.e. not shared between emotions). The [AmbImprov > AmbChrom] contrast showed activations in the right superior temporal lobe (BA 22 and BA 21, Wernicke’s Area homologue) and temporal pole (BA 38), but these activations were not seen in [PosImprov > PosChrom] or [NegImprov > NegChrom]. Recent evidence indicates that the right hemisphere language areas are involved in resolving lexical ambiguity^28^, therefore these results suggest that Wernicke’s area right hemisphere homolog may be involved in processing musical ambiguity as well.

To further examine the effect of emotion on improvisation, we looked at the between-emotion contrasts (ex. [PosImprov > NegImprov]). These contrasts revealed significant differences between emotion blocks, most notably in areas known for their involvement in emotional processing. The most extensive differences were seen when negative and ambiguous improvisation were compared to positive improvisation. [AmbImprov > PosImprov] and [NegImprov > PosImprov] contrasts both revealed heightened activity in the right insula (BA 13 and 47) and right anterior cingulate cortex (BA 32), as well as activation in the right parietal cortex (BA 40), bilateral middle temporal lobes (BA 22), and bilateral middle frontopolar prefrontal cortex (FPPFC, BA 10) (Fig. 4, Table 2). Positive improvisation showed increased cerebellar activity compared to negative improvisation ([PosImprov > NegImprov]), and markedly increased activity in the left hippocampus and amygdala and right parahippocampal gyrus when compared to ambiguous improvisation ([PosImprov > AmbImprov]) (Fig. 4, Table 2).

In addition to the differences described above, we observed differences between negative and ambiguous improvisation trials. The contrast [NegImprov > AmbImprov], showed increased activity in the right anterior cingulate (BA 9), left angular gyrus and supramarginal gyri (BA 39 and 40) and the right hippocampus. The converse contrast ([AmbImprov > NegImprov]) showed a relative increase in activity in the right cerebellum (Vermis, VII), bilateral Heschl’s gyrus (BA 41), and left primary motor areas (BA 4) during ambiguous improvisation. The observed activity in the cerebellum and primary motor area may be due to the increased number of notes played (increased movement) during ambiguous improvisation compared to negative improvisation. For between-emotion viewing contrasts, there were no significant clusters between any of the emotion and viewing conditions, [AmbView > PosView], [PosView > AmbView], [PosView > SadView], [PosView > NegView], or [NegView > AmbView]. There were small ( < 16voxel) significant clusters for the contrast, [AmbView > NegView], in the insula, caudate and cerebellum (Cerebellum—6). The minimal number of significant differences between viewing conditions suggests that widespread differences between emotion improvisation conditions cannot be attributed to the emotional valence of the cue photographs. The photographs, rather than eliciting strong emotions, were serving as emotional ‘guideposts’ for the subjects.

### Functional Connectivity: Psycho-Physiological Interaction (PPI) Analysis

We originally hypothesized that functional connectivity between brain areas that support emotion processing and regions of the brain that support improvisation would be modulated by emotional intent. Thus, we looked for regions of the brain that support emotion processing to use as seeds in a PPI analysis. Due to the prominent role of the amygdala and anterior insula in previous investigations of music-evoked emotions^29^ and more general emotional experience^30^, the left amygdala and left anterior insula clusters from the between-emotion contrasts were selected as seed regions for PPI analysis. Prefrontal regions, such as the DLPFC, were not chosen as seeds related to emotion processing, as these regions, even when they appear in studies of emotion processing, are typically interpreted as supporting cognitive processes that are secondary to emotional experience or responsive to task demands, rather than primary to a given emotional experience^30^. Within-emotion contrasts in the amygdala PPI analysis demonstrated lower connectivity during positive improvisation than during the positive chromatic condition between the left amygdala and the cerebellum (Table 3a). A decrease was observed during negative improvisation compared to the negative chromatic condition in amygdala connectivity with the right inferior frontal gyrus and left postcentral gyrus areas (Table 3a, Fig. 5a).

In the between-emotion contrasts, overall greater connectivity in the positive improvisation condition than in the negative improvisation condition was observed between left amygdala and the superior medial and superior frontal gyri, the anterior cingulate, the supramarginal gyrus, and the inferior parietal sulcus (Table 3a, Fig. 5a). Lower effective connectivity in the positive improvisation condition than in the positive chromatic condition was observed between the left insula and superior and middle frontal gyri, precentral and postcentral gyri, and the supramarginal gyrus, and greater effective connectivity was observed between the left insula and primary visual cortex. We also observed lower effective connectivity between the left insula and middle frontal gyrus, and the left insula and inferior parietal lobule, with greater effective connectivity between left insula and superior medial gyrus. When considering the between-emotion contrast, greater effective connectivity was observed between the left insula and the rolandic operculum during positive improvisation than during negative improvisation. Greater effective connectivity was also observed between the left insula and a midbrain region that includes the substantia nigra during negative improvisation compared to positive improvisation (Table 3b, Fig. 5b).

## Discussion

Here we examined the interaction between emotional expression and creativity in an ecologically valid context. MIDI performance analysis revealed that pianists were consistently using musical features such as mode, note density, duration and range to distinguish positive, ambiguous and negative emotions. These results match findings from a previous study of jazz improvisation in response to visual emotional cues, done outside of a scanner^13^, and this similarity indicates that the pianists were improvising comfortably within the fMRI scanner. Furthermore, the pianists were playing on a keyboard with full size keys, and they could comfortably hear their playing in real time. It could be argued that by playing (and simultaneously hearing) their emotionally expressive improvisations, the subjects were inducing emotions in themselves through a positive auditory feedback loop. While this is possible, a feedback effect may occur whether pianists are able to hear themselves or not, as there is evidence for strong auditory-motor coupling in professional pianists. Several studies have shown similar neural activations between pianists performing on a mute vs. sounding piano^31^,^32^. If hearing their improvisations increased the musicians’ emotional arousal, this increase is in line with the aims of the current study. When we examined the functional neuroimaging results, we found that the creative expression of emotions through music may engage emotion-processing areas of the brain in ways that differ from the perception of emotion in music. We also observed a functional network involved in creative performance, and the extent of activation and deactivation in this network was directly modulated by emotional intent. Our viewing controls showed that there were few significant differences between neural activity in response to any of the visual cues, therefore the differences between improvisation conditions are the result of the creative expression of emotion through music, rather than a direct response to the visual stimuli. These results highlight that creativity is context-dependent, and emotional context critically impacts the neural substrates of artistic creativity.

Our study suggests that expressing emotion through music engages limbic and prefrontal areas of the brain in a distinctive manner not previously observed in other studies of music perception. Although insular and hippocampal activity has been observed in response to joyful music^29^,^33^,^34^,^35^, we observed an opposite pattern of more robust insular and hippocampal activity during negative improvisation. A possible explanation for this difference is that music perception generally activates the network involved in creativity (the precuneus, MPFC, DLPFC, etc.)^36^,^37^. The deactivation of this system during creative tasks may change how the neural networks involved in emotion are recruited during performance when compared to perception. These differences are critical, as they may relate in part to the fundamentally different processes of observing an emotional state in somebody or something else and personally experiencing that emotional state. Clearly, these processes are linked to one another through empathy and experience, yet it is possible that the personal experience of an emotion and behavioral changes caused by the need or desire to express that emotion (through art or otherwise) are more compelling than observation of that emotion alone. Future studies of the key differences between emotional expression and perception are needed so that we may arrive at a more comprehensive neural model of emotion.

Our random effects analyses indicate that there is an essential functional network involved in creative expression, and that this network is modulated by emotional intent. Several studies have shown that spontaneous musical improvisation induces deactivations in the DLPFC, angular gyrus, and precuneus, and activations in the SMA and perisylvian language areas^20^,^21^,^22^,^38^,^39^,^40^. We observed changes in all of these areas in response to the three different emotional intents. Most strikingly, DLPFC deactivation was much more pronounced during positive improvisation when compared to negative and ambiguous improvisation. A decrease in prefrontal cortical activity (hypofrontality) has been implicated in various altered states of consciousness and flow states^24^,^25^. Furthermore, while we did not observe activations in the SMA during positive improvisation, it was active during negative and ambiguous improvisation, primarily in the left hemisphere (expected due to the pianists’ use of their right hand while playing). Based on the MIDI data analysis, it is unlikely that this SMA activity simply relates to increased motor activity – performers were, on average, playing fewer notes during negative and ambiguous improvisations when compared to positive improvisation. The SMA is often active during tasks involving continuous monitoring of the appropriateness of motor output, including rhythmic tapping and complex sequential motor tasks^41^. The observed hypofrontality and lack of SMA activity may indicate that positive improvisation induces a deeper state of flow than negative and ambiguous improvisation.

Since listening to sad music is often pleasurable^42^, we originally predicted that we would observe increased hypofrontality during negative improvisation as well as positive improvisation (when both were compared to ambiguous improvisation). The combination of our random effects and functional connectivity analyses present an intriguing alternative: positive and negative improvisation may be pleasurable for different reasons. Negative improvisation showed increased functional connectivity between the insula and the substantia nigra (a dopaminergic reward area) when compared to positive improvisation. This type of connectivity could imply an increased binding of visceral awareness^43^, and this finding is consistent with a recent report outlining the rewarding properties of sad music^42^. Music-related rewards include the ability to experience or express emotions without any “real-life” costs. This is a reward that could certainly be maximized during music improvisation, and that could be expected to drive activity in dopaminergic regions such as the substantia nigra. The rewarding aspects of sad music may also be contingent on maintaining a degree of cognitive distance from the artwork^3^. This interpretation is consistent with increased activation in areas responsible for cognitive control and self-monitoring, primarily the SMA and frontal polar prefrontal cortex, during negative improvisation when compared to positive improvisation. While further work is needed to clarify this complex relationship, it is plausible that the pleasure or satisfaction derived from happy and sad music may be mediated through different neurobiological systems. While positive emotional targets enable more widespread hypofrontality and deeper flow states during spontaneous creativity, negative emotional targets may be more closely linked to a stronger visceral experience and greater activity in reward processing areas of the brain during improvisation.

In summary, this study shows that the impulse to create emotionally expressive music may have a basic neural origin: emotion modulates the neural systems involved in creativity, allowing musicians to engage limbic centers of their brain and enter flow states. The human urge to express emotions through art may derive from these widespread changes in limbic, reward, and prefrontal areas during emotional expression. Within jazz improvisation, certain emotional states may open musicians to deeper flow states or more robust stimulation of reward centers. The creative expression of emotion through music may involve more complex mechanisms by which the brain processes emotions, in comparison to perception of emotion alone. Additional studies of how emotional state modulates creativity in non-artistic domains such as decision-making and social interactions are needed. Future studies could also examine whether there is an effect of gender on emotional expression through music, and whether the neural results are altered if subjects use both hands during improvisation. This study examines just one of many possible factors that could influence the neural underpinnings of human creativity, and there is huge scope for investigation. Further understanding how emotion influences creativity in both artistic and non-artistic contexts will be crucial for the derivation of a more comprehensive and accurate neural model of human creativity.

## Methods

### Stimuli Selection

**Figure 1:**
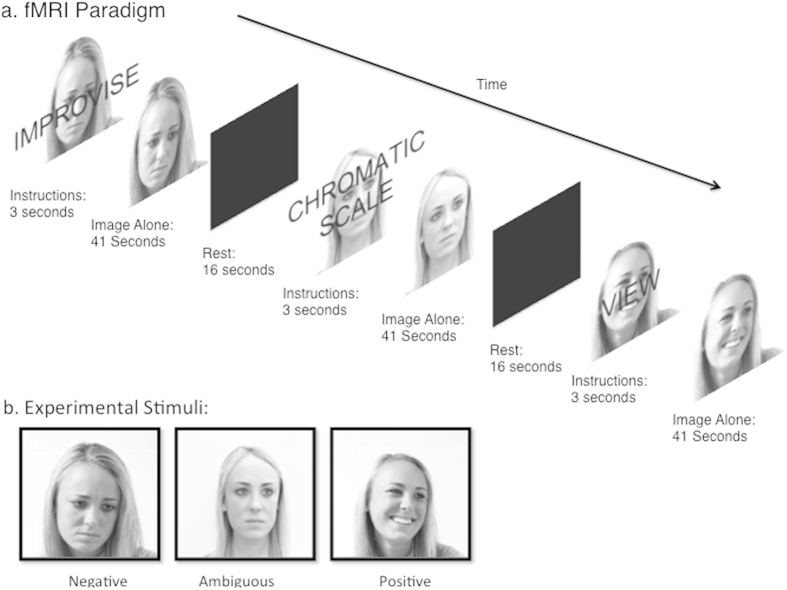
fMRI Paradigm and Photographs. Used as Visual Stimuli (a) Schematic showing fMRI stimulus and instruction presentation paradigm and (b) Photographs representing positive, ambiguous and negative emotions. Photographs were shot indoors in black and white with a 50 mm lens at f16 using a Nikon D700 digital SLR camera.

**Figure 2:**
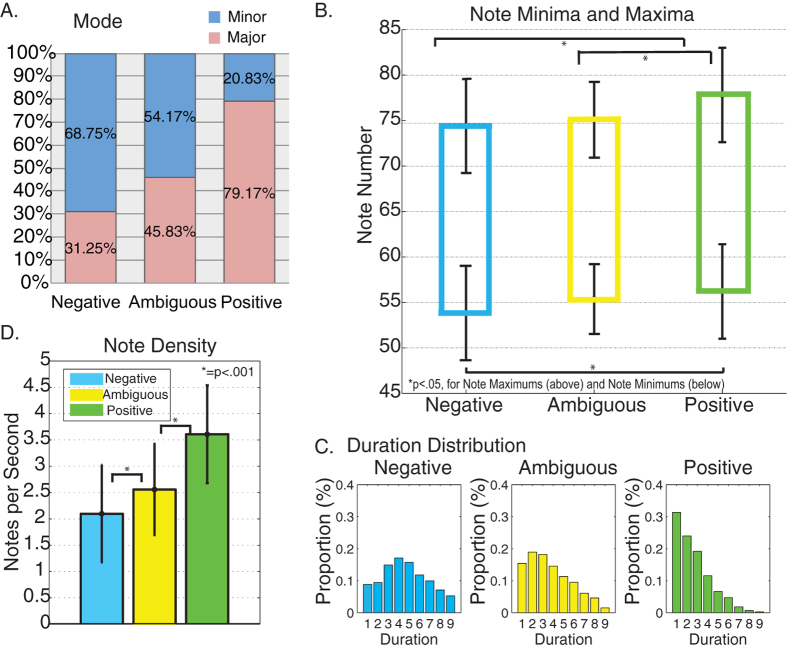
MIDI Analysis Results. Differences in proportion of major to minor keys, note minima and maxima, distributions of note durations, note density.

**Figure 3:**
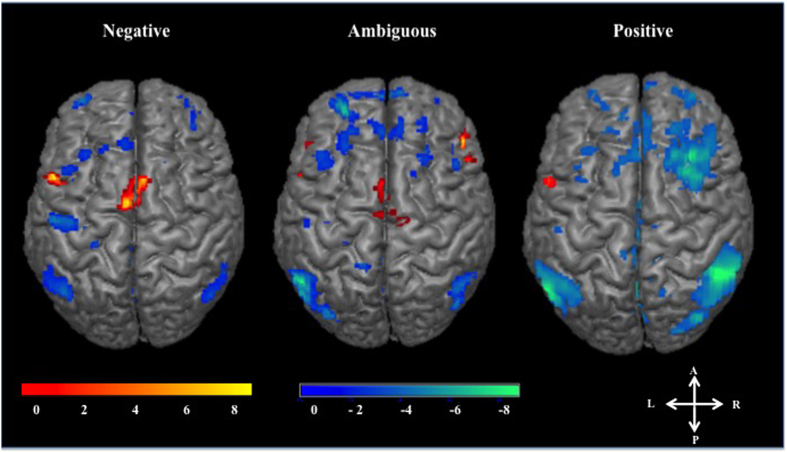
Within-Emotion Contrasts. Three-dimensional surface projections of activations (ex. PosImprov > PosChrom) and deactivations (ex. PosChrom > PosImprov) during improvisation for different emotion conditions. Results are from a random effects model, p < 0.005 with a 10 voxel cluster threshold. Improvisation was associated with perisylvian language area activations and supplementary motor area activations across emotions, as well as deactivations in the DLPFC, angular gyrus, and precuneus. The scale bar shows the range of t-scores; the axes demonstrate anatomic orientation. Abbreviations: A, anterior; P, posterior; R, right; L, left.

**Figure 4:**
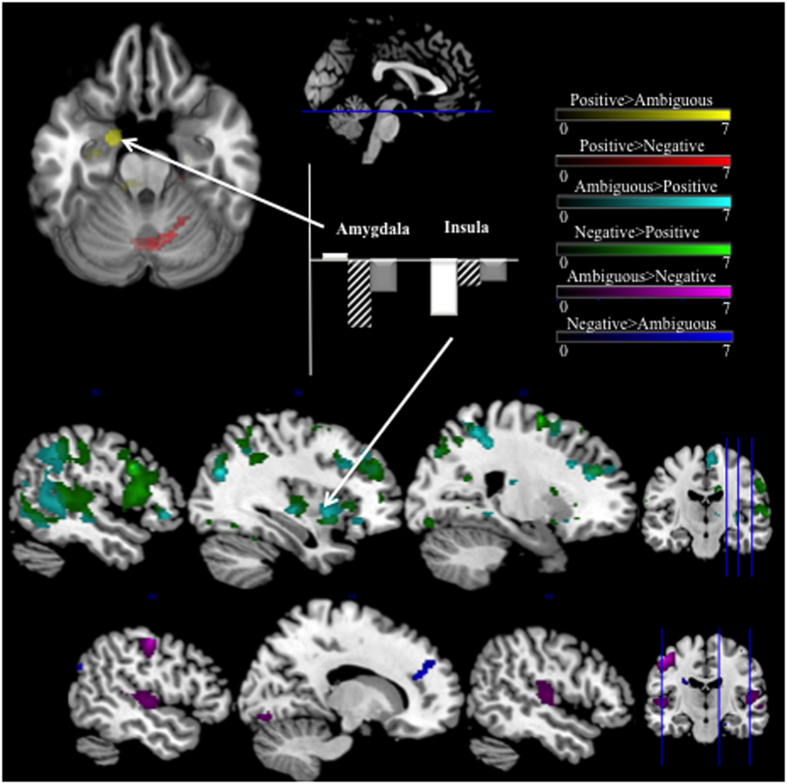
Between-Emotion Contrasts. Top row shows an axial slice rendering of contrasts between positive and ambiguous improvisation (PosImprov > AmbImprov—Yellow) and positive and negative improvisation (PosImprov > NegImprov—Red). Specific to positive improvisation, we observed increased activation in the left amygdala and right cerebellum. The middle row shows sagittal slice renderings of contrasts between ambiguous and positive improvisation (AmbImprov > PosImprov—Cyan) and negative and positive improvisation (NegImprov > PosImprov—Green). These contrasts showed widespread differences, primarily in right hemisphere superior temporal lobe, right insula, anterior cingulate cortex and superior parietal lobe. The third row shows sagittal Axial slice renderings of contrasts between negative and ambiguous improvisation (AmbImprov > NegImprov—Violet, NegImprov > AmbImprov—Blue). Primary differences between ambiguous and negative Improvisation were increased anterior cingulate activation during negative, and increased right superior temporal lobe and primary motor area activations during ambiguous. Sagittal sections show axial slice location. Coronal sections show sagittal slice locations. All results are from random effects models, *p* < 0.005, 10 voxel cluster threshold. Bar graphs indicate percent signal change at cluster maxima, plotted using rfxplot, Glascher 2009). White = positive improvisation, Dashed = ambiguous improvisation, Grey = negative improvisation.

**Figure 5:**
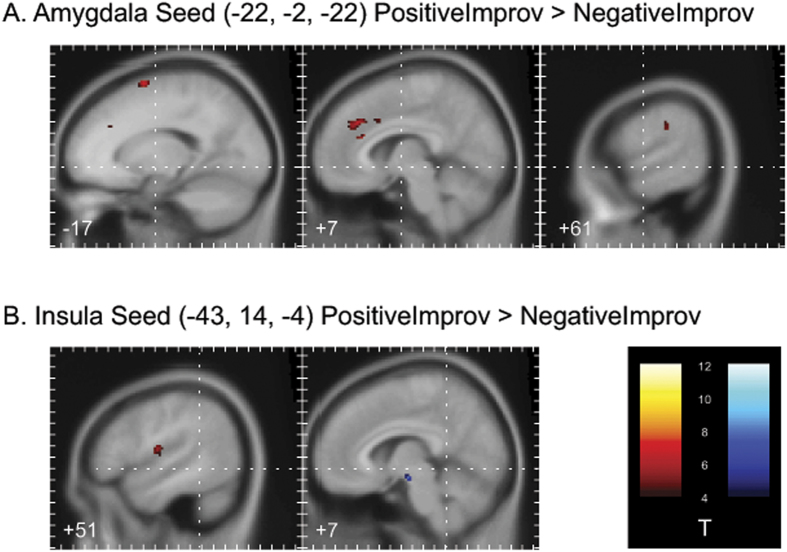
PPI Results. (**A**) Regions showing significantly higher (in red) effective connectivity with the left amygdala (x = −22, y = −2, z = −22) during positive compared to negative improvisation [PosImprov > NegImprov contrast in gPPI analysis]. (**B**) Regions showing significantly higher (in red) or lower (in blue) effective connectivity with the left insula (x = −43, y = 14, z = −4) during positive compared to negative improvisation [PosImprov > NegImprov contrast in gPPI analysis]. Contrasts are thresholded at p < 0.001 (uncorrected) with a minimum cluster size of 20 voxels. Panels are plotted in the sagittal plane, with the x-coordinate of each panel indicated in the lower left-hand corner of each slice. Tick marks on the y-axis of each panel indicate differences of 10 mm in the y dimension of the image space. Tick marks on the x-axis of each panel indicate differences of 10 mm in the z dimension of the image space. Dashed lines through the center of each slice converge on the origin of the image in the axial and coronal planes. All coordinates are presented in MNI space.

In a pre-experiment survey, three photographs of an actress expressing a basic emotional valence (positive, ambiguous and negative) were chosen as emotional cues for the current study (Fig. 1b). “Ambiguous” was defined as a neither positive nor negative rating in valence and arousal^44^. These stimuli were first developed for a previous, behavioral study of emotional improvisation^13^, and were intended to represent an emotion with minimal distractions, and without eliciting a strong emotional reaction from perceivers. Photos showed an actress from the collarbone upward, looking away from the camera, and photos were desaturated so there would be no color cues. We developed these visual cues for emotions in order to avoid potential confounds of linguistic labels^45^.

11 males and 9 females (mean age = 32 ± = 17s.d.), from the Johns Hopkins University community rated a selection of images on a visual analog scales based on Russell’s circumplex model^36^,^44^, and results were coded on a nine point scale (0–9, Negative-Positive). We calculated a one-way ANOVA with factors Emotion (Negative, Ambiguous, Positive). Tukey’s honestly significant difference criterion was used for post hoc comparisons. A significant main effect of Emotion [F(1, 2) = 110.87, p < 0.001] was observed. Mean ratings for the stimuli: Negative, mean = 2.95, s.d. = 1.09, Ambiguous, mean = 4.3, s.d. = 0.80, Positive, mean = 7.5, s.d. = 1.05. A full description of our stimuli pre-testing is available in McPherson *et al.*^13^. Informed consent was obtained in writing from all subjects, and all experimental procedures were approved by the Johns Hopkins University School of Medicine Institutional Review Board. All experimental procedures were carried out in accordance with the approved guidelines.

### Musical Performance Analysis

We analyzed the MIDI (Musical Instrument Digital Interface) piano output obtained during fMRI scanning using measures of salient musical features including note density, note duration distribution, note maxima and minima, mode, and key. These measures were compared for the chromatic scales and improvisations created in response to the different emotional targets. These results were calculated using the MIDI Toolbox^46^, and a complete explanation of the calculation of these features can be found in Eerola and Toiviainen, 2004.

Note density is a measure of notes per second, and for monophonic compositions can be used as an indication of tempo (higher note densities generally correspond with faster tempos). Note maxima and minima indicate the highest and lowest pitch, respectively, played in a given musical segment. For note density, maxima and minima, we calculated a one-way ANOVA with the within-subject factor Emotion (Negative, Ambiguous, Positive) for both improvisation and chromatic scale trials. Tukey’s honestly significant difference criterion was used for post hoc comparisons.

The duration distribution function of the MIDI Toolbox returns the percentage of notes that fall into nine different logarithmically organized bins (note length categories). Length categories are defined as a unit of beats. We set our MIDI tempo so that 1 beat = 0.5 s (quarter note = 120 Beats Per Minute (BPM)). Therefore, bin 1 = 1/8 s, bin 3 = ¼ s, bin 5 = ½ s, bin 7 = 1 s, and bin 9 = 2 s. The relationship between bin 1 and bin 9 is proportional to the relationship between a sixteenth note and a whole note. We compared corresponding duration distribution bins using two-sample Kolmogorov-Smirnov tests.

Key (tonal center) and mode (major vs. minor) were calculated using the Krumhansl & Schmuckler (K-S) key-finding algorithm, which uses the pitch class distribution of a piece (weighted according to duration) to return a key profile for the piece^46^. We used the K-S key finding algorithm to determine the best fit for each entire 44 s improvisation. Mode and key calculations were confirmed by the authors through a visual inspection of the scores.

### Functional Neuroimaging Testing

#### Subjects

Twelve professional jazz pianists (11 male, 1 female; mean age = 39.9 ± 15.8) participated in the study. All subjects had been performing piano professionally for over 5 years (mean yrs performing professionally = 18.35 ± 13.28). Subjects were recruited as they became available, without an a-priori regard to balancing by gender. None of the subjects reported histories of neurologic, auditory or psychiatric disorders. Informed consent was obtained in writing for all subjects, and the research protocol was approved by the Johns Hopkins School of Medicine Institutional Review Board.

#### Experimental Design

A block-design imaging paradigm was used to assess the effect of emotional intent on musical creativity (Fig. 1a). Rest blocks were 16 seconds in duration, and test blocks were 44 seconds in duration. While in the scanner, the pianists were shown the three selected photographs of an actress representing, ‘Positive’, ‘Negative’, and ‘Ambiguous’ emotions. At the beginning of the presentation of each image, subjects were given a simultaneous matching visual and auditory cue instructing them to respond in a specific way to the image. This cue lasted three seconds. The cues were: “View”, “Chromatic Scale” and “Improvise”. Subjects were instructed to simply fixate on the image and keep their eyes open during the entire ‘View’ condition. The chromatic scale condition was designed to assess neural activity during a highly constrained, non-creative and non-emotional musical motor task. For the chromatic condition, subjects were told to play an ascending and descending chromatic scale over the entire range of the keyboard. Pianists were instructed to make this chromatic scale the same tempo regardless of the picture they were viewing. Before scanning began, pianists were familiarized with the target tempo, approximately eighth note = 180 BPM (three notes per second), and were instructed to keep this tempo consistent between blocks. For the improvise condition, subjects were instructed to improvise a composition that they felt best represented the emotion expressed by the images. Improvisation was unrestricted melodically, harmonically, and rhythmically, but the subjects were instructed to play monophonically (one note at a time) using their right hand. Pianists were restricted to using their right hand while improvising and playing chromatic scales due to space considerations on the scanner piano. Stimuli were presented in a pseudorandom order, and there were 24 test blocks per subject, with 8 per emotion (4 Improvisation, 2 Chromatic and 2 View). Subjects were asked to keep their eyes open during the entire experiment, even rest blocks, and to refrain from moving their head or any other part of their body other than their right hand.

#### Procedure

During scanning, subjects used a custom-built non-ferromagnetic piano keyboard (MagDesign, Redwood, CA) with thirty-five full-size plastic piano keys. The piano keyboard was placed on the subject's lap in supine position, while their knees were elevated with a bolster. A soft velcro square was placed on Middle C of the piano, allowing subjects to orient their hand without viewing the keyboard. Subjects were visually monitored to ensure that they did not move their left (non-playing) hand, head, trunk, or other extremities during performance. Before the experiment commenced, subjects were given time to find a comfortable playing position and familiarize themselves with the keyboard and environment. Subjects also had a trial run to test sound levels, make final keyboard placement adjustments, and practice the paradigm before scanning began. Data from these test blocks were discarded, and the test block was repeated if the subject requested more time to become comfortable in the scanner.

The scanner keyboard had MIDI output, which was sent to a Macintosh MacBook Pro laptop computer running the Logic Express 8 sequencing environment (Apple Inc., Cupertino, CA). Piano sound output was routed back to the subject via in-ear electrostatic earspeakers (Stax, Saitama, Japan). In addition to the electrostatic earspeakers, subjects wore additional ear protection to minimize background scanner noise. For each subject, ear speaker volume was set to a comfortable listening level that could be easily heard over the background scanner noise. A double mirror mounted on the head coil above the subject's eyes allowed them to view a rear projection screen behind the scanner bore. The stimuli and instructions were presented with EPrime^47^.

#### Image acquisition

All scans were performed at the F.M. Kirby Research Center for Functional Brain Imaging at the Kennedy Krieger Institute of Johns Hopkins University. Blood oxygen level dependent imaging (BOLD) data and T1-weighted anatomical images were acquired using a 3-Tesla whole-body scanner (Philips Electronics, Andover, MA) using an sixteen-channel head coil and a gradient-EPI sequence. The following scan parameters were used: TR = 2000 ms, TE = 30 ms, flip-angle = 75 degrees, field of view 216.000 × 128.000 × 240.000 mm, 32 parallel axial slices covering the whole brain, 4 mm thickness (3 × 3 mm in-plane resolution). 720 volumes were acquired for each subject.

#### fMRI Analysis

Standard preprocessing steps were completed in SPM8, including realignment to the first volume of the run, coregistration with a participant's T1-weighted structural image, indirect normalization of the structural image to template space, propagation of normalization parameters to coregistered functional images, and smoothing with an 8 mm FWHM kernel. A first-level general linear model was estimated for each subject using ten regressors, one for rest and one for each experimental condition combination—emotion (positive, negative, ambiguous—Pos, Neg and Amb) and task (view, chromatic scale, improvisation—View, Chrom and Improv). Each regressor was convolved with a standard hemodynamic response function. Design matrices also included covariates of non-interest, which consisted of motion parameters calculated during the realignment stage and mean signal intensity for the run. Between-emotion (e.g. [PosImprov > NegImprov]) and within-emotion (e.g. [PosImprov > PosChrom]) contrasts were estimated for each subject. Contrasts were then entered into a second-level random-effects model using a one-sample t-test. Random-effects analyses take into account inter-subject variability, and therefore can be generalized to a broader population. Contrasts were thresholded at an uncorrected *p* value of 0.005 with a minimum voxel extent of 10 voxels. Analysis of average effect sizes was completed using the rfxplot toolbox^48^.

#### PPI Analysis

Psycho-physiological interaction (PPI) analysis can be used to identify task-dependent changes in effective connectivity between a seed region and other regions in the brain^49^. We used the generalized psycho-physiological interaction (gPPI) toolbox^50^ to examine differences in the networks of brain areas that exhibited functional connectivity with emotion-specific brain areas during improvisation conditions with different emotional intention. Seed regions for this analysis were derived from emotional condition contrasts estimated in the primary analysis. Significant shifts in functional connectivity in an emotional condition for each seed region were identified by applying inclusive masking for within-emotion (i.e. [PosImprov > PosChrom]) and between-emotion (i.e. [PosImprov > NegImprov]) contrasts, with a minimum voxel extent of 20 voxels and p < 0.001 significance threshold, uncorrected.

## Additional Information

**How to cite this article**: McPherson, M. J. *et al.* Emotional Intent Modulates The Neural Substrates Of Creativity: An fMRI Study of Emotionally Targeted Improvisation in Jazz Musicians. *Sci. Rep.* **6**, 18460; doi: 10.1038/srep18460 (2016).

## Figures and Tables

**Table 1:** Within-Emotion Contrasts.

Activations		Negative Improvisation				Cluster Size					Cluster Size
Region	BA	*Left Hemisphere*					*Right Hemisphere*				
		t-score	x	y	z		t-score	x	y	z	
SMA	6	5.47	−4	0	66	145*	4.61	4	14	64	145*
Precentral Gyrus	9	6.1	−52	12	32	42	—	—	—	—	—
*Perisylvian Language Areas*											
IFG-P-OP	44	5.07	−54	10	16	48	—	—	—	—	—
IFG-P-Tr	45	4.57	−54	10	20	11	—	—	—	—	—
Deactivations		Negative Improvisation									
Region	BA	*Left Hemisphere*					*Right Hemisphere*				
		t-score	x	y	z	Cluster Size	t-score	x	y	z	Cluster Size
Hippocampus		−3.32	−30	−36	−8	19	−3.29	32	−16	−14	13
*Prefrontal Cortex*											
FPPFC	10	−4.77	−34	58	2	35	−3.88	40	50	4	30
DLPFC	9	−3.39	−30	30	38	14	—	—	—	—	—
Secondary Clusters	9	−4	−44	18	40	13	—	—	—	—	—
DMPFC	8	−3.93	−6	36	48	26	—	—	—	—	—
	9	−3.46	−4	50	24	10	—	—	—	—	—
*Cingulate Gyrus*											
MCC	31	−5.29	−2	−42	36	197*	−4.7	4	−42	32	197*
*Parietal Lobe*											
Superior Parietal	40	−3.97	−54	−58	38	129	−4.09	48	−64	46	40
AG	39	−4.12	−52	−60	24	83	−3.95	48	−66	42	11
Precuneus	7	−4.12	−8	−64	38	86*	−3.72	6	−64	38	86*
Activations	Ambiguous Improvisation										
Region	BA	*Left Hemisphere*					*Right Hemisphere*				
		t-score	x	y	z	Cluster Size	t-score	x	y	z	Cluster Size
SMA	6	4.59	−2	12	60	24	—	—	—	—	—
Secondary Clusters	6	3.85	−2	−8	62	37	—	—	—	—	—
*Perisylvian Language Areas*											
IFG-P-OP	44	4.67	−54	8	12	64	3.96	52	16	12	21
IFG-P-Tr	45	6.63	−50	18	16	19	4.12	54	18	15	33
STG	22	—	—	—	—		5.89	54	−26	0	30
*Deactivations*	Ambiguous Improvisation										
Region	BA	*Left Hemisphere*					*Right Hemisphere*				
		t-score	x	y	z	Cluster Size	t-score	x	y	z	Cluster Size
Hippocampus							−5.35	28	−16	−20	52
*Prefrontal Cortex*											
FPPFC	10	−5.14	−28	54	18	102*	−5.13	10	64	14	102*
DLPFC	9	−3.87	−36	22	40	49	−3.98	46	24	40	10
Secondary Clusters	9	−3.87	−38	34	42	24	—	—	—	—	—
DMPFC	8	—	—	—	—	—	−4.42	24	22	42	38
	9	−4.08	−14	40	20	15	—	—	—	—	—
*Cingulate Gyrus*											
MCC	31	−3.84	2	−38	36	70*	−5.07	−2	−38	36	70*
ACC	32						−5.69	8	38	28	62
*Parietal Lobe*											
Superior Parietal	40	−7.81	−54	−56	42	136	−4.9	50	−54	48	11
AG	39	−5.59	−52	−66	32	74	−4.97	46	−70	34	21
Precuneus	7	−3.56	−4	−60	42	160*	−4.66	8	−62	44	160*
Activations	Positive Improvisation										
Region	BA	*Left Hemisphere*					*Right Hemisphere*				
		t-score	x	y	z	Cluster Size	t-score	x	y	z	Cluster Size
Precentral Gyrus	9	5.24	−56	10	34	78	—	—	—	—	—
*Perisylvian Language Areas*											
IFG-P-OP	44	3.16	−52	10	22	20	—	—	—	—	—
Deactivations	Positive Improvisation										
Region	BA	*Left Hemisphere*					*Right Hemisphere*				
Hippocampus		−9.8233	−26	−36	−2	236					
*Prefrontal Cortex*											
FPPFC	10	−4.01	−25	60	2		−4.85	32	56	2	52
DLPFC	9	−3.16	−30	32	42	13	−5.62	38	24	40	234
Secondary Clusters	9	—	—	—	—	—	−7.24	32	26	46	190
DMPFC	8	−4.31	−2	30	54	107*	−3.93	2	30	52	107*
	9	−3.81	−6	46	20	166*	−4.31	6	46	38	166*
*Cingulate Gyrus*											
MCC	31	−5.32	−2	−44	34	470*	−5.32	4	−38	34	470*
ACC	32	−3.89	−8	42	16	71*	−3.81	6	42	16	71*
*Parietal Lobe*											
Superior Parietal	40	−6.86	−56	−58	30	258	−7.43	60	−48	40	627
AG	39	−7.01	−58	−88	28	229	−3.51	58	−58	24	64
Precuneus	7	−5.82	−2	−64	36	569*	−7.47	10	−64	32	569*

All coordinates are described according to the Montreal Neurological Institute system, and were obtained through a random effects (uncorrected, p < 0.005, 10 voxel cluster threshold) analysis of data.Abbreviations: SMA, supplementary motor area; IFG-P-Op, inferior frontal gyrus pars opercularis; IFG-P-Tr, inferior frontal gyrus pars triangularis; STG, superior temporal gyrus; FPPFC, frontopolar prefrontal cortex; DLPFC, dorsolateral prefrontal cortex; DMPFC, dorsomedial prefrontal cortex; MCC, middle cingulate cortex; ACC, anterior cingulate cortex; AG, angular gyrus.

**Table 2:** Between-Emotion Contrasts.

Activations	NegativeImprov > PositiveImprov					Cluster Size					Cluster Size
Region	BA	*Left Hemisphere*					*Right Hemisphere*				
		t-score	x	y	z		t-score	x	y	z	
SMA	6	—	—	—	—	—	5.28	6	−12	72	277
ACC	32	—	—	—	—	—	4.71	16	34	26	
Insula	13	3.8	−40	8	−4	17	3.41	36	12	−8	13
	47	—	—	—	—	—	3.83	40	24	2	25
*Frontal Lobe*											
FPPFC	10	3.7	−40	40	14	10	4.11	26	46	30	
	9	4.19	−42	12	38	14	4.55	38	20	34	9
*Perisylvian Language Areas*											
MTG	21	3.98	−64	−40	0	22	5.36	60	−42	−2	62
STG	22	4.24	−64	−38	2	19	4.48	56	−44	2	54
*Parietal Lobe*											
Precuneus	7	—	—	—	—	—	5.41	4	−44	46	136
Angular Gyrus	7	—	—	—	—	—	3.21	38	−58	50	19
Superior Parietal	40	4.29	−64	−32	32	122	5.64	44	−46	44	264
Activations	AmbiguousImprov > PositiveImprov										
Region	BA	*Left Hemisphere*					*Right Hemisphere*				
		t-score	x	y	z	Cluster Size	t-score	x	y	z	Cluster Size
SMA	6	4.33	−2	2	60	225*	4.59	20	−2	64	225*
Insula	13	—	—	—	—	—	4.4	36	22	2	62
Insula	47	—	—	—	—	—	4.63	38	22	0	31
*Cingulate Lobe*											
ACC	32	—	—	—	—	—	4.36	16	34	36	30
MCC	24	—	—	—	—	—	6.16	8	4	38	85
*Frontal Lobe*											
Precentral gyrus	9	—	—	—	—	—	7.2	54	12	32	235
FPPFC	10	4.4	−26	54	4	27	4.7	34	44	28	60
*Perisylvian Language Areas*											
STG	22	4.14	−64	−42	2	75	6.04	66	−14	4	124
IFG-P-OP	44	7.67	−44	16	8	17	6.6	52	12	8	125
IFG-P-Tr	45	5.52	−46	18	6	29	5.56	48	22	10	121
*Parietal Lobe*											
Angular Gyrus	40	—	—	—	—	—	4.59	36	−56	48	337
Precuneus	7	—	—	—	—	—	4.63	20	−74	44	170
Activations	PositiveImprov > NegativeImprov										
Region	BA	*Left Hemisphere*					*Right Hemisphere*				
		t-score	x	y	z	Cluster Size	t-score	x	y	z	Cluster Size
Cerebellum—VI		4.04	−8	−64	−26	113	4.07	10	−64	−24	157
Parahippocampal gyrus	35	—	—	—	—	—	3.51	22	−24	−22	7
Activations	PositiveImprov > AmbiguousImprov										
Region	BA	*Left Hemisphere*					*Right Hemisphere*				
		t-score	x	y	z	Cluster Size	t-score	x	y	z	Cluster Size
Amygdala		6.18	−22	−2	−22	59	—	—	—	—	—
Hipocampus		3.9	−32	−26	−14	95	—	—	—	—	—
Parahippocampal gyrus	28	—	—	—	—	—	3.32	22	−14	−24	11
Activations	AmbiguousImprov > NegativeImprov										
Region	BA	*Left Hemisphere*					*Right Hemisphere*				
		t-score	x	y	z	Cluster Size	t-score	x	y	z	Cluster Size
Vermis—VII		—	—	—	—	—	3.94	0	−72	−26	17
Primary Motor Area	4	10.05	−46	−14	54	238	—	—	—	—	—
STG	22	4.69	−50	−14	4	45	5.87	56	−8	−2	85
Heschel's Gyrus	41	3.63	−48	−20	10	65	4.06	48	−20	10	47
Activations	NegativeImprov > AmbiguousImprov										
Region	BA	*Left Hemisphere*					*Right Hemisphere*				
		t-score	x	y	z	Cluster Size	t-score	x	y	z	Cluster Size
Hippocampus		—	—	—	—	—	4.8	30	−40	−2	12
ACC	9	—	—	—	—	—	5.74	14	40	24	19
Angular Gyrus	39	3.69	−48	−72	30	19	—	—	—	—	—
SMG	40	3.76	−60	−56	28	12	—	—	—	—	—

All coordinates are described according to the Montreal Neurological Institute system, and were obtained through a random effects (uncorrected, p < 0.005, 10 voxel cluster threshold) analysis of data. Abbreviations: SMA, supplementary motor area; IFG-P-Op, inferior frontal gyrus pars opercularis; IFG-P-Tr, inferior frontal gyrus pars triangularis; STG, superior temporal gyrus; FPPFC, frontopolar prefrontal cortex; DLPFC, dorsolateral prefrontal cortex; DMPFC, dorsomedial prefrontal cortex; MCC, middle cingulate cortex; ACC, anterior cingulate cortex; AG, angular gyrus.

**Table 3:** PPI Results.

**A) Amygdala Seed (−22, −2, −22)**										
Region	PositiveImprov > PositiveChromatic									
	*Left Hemisphere*					*Right Hemisphere*				
	t-score	x	y	z	Cluster Size	t-score	x	y	z	Cluster Size
Cerebellum	−5.3549	−8	−38	−22	423	—	—	—	—	—
	NegativeImprov > NegativeChromatic									
	*Left Hemisphere*					*Right Hemisphere*				
	t-score	x	y	z	Cluster Size	t-score	x	y	z	Cluster Size
Inferior Frontal Gyrus (pars orbitalis)	—	—	—	—	—	−5.7533	42	46	−4	27
Postcentral Gyrus	−6.6215	−56	−12	44	39	—	—	—	—	—
	PositiveImprov > NegativeImprov									
	*Left Hemisphere*					*Right Hemisphere*				
	t-score	x	y	z	Cluster Size	t-score	x	y	z	Cluster Size
Superior Medial Gyrus	7.9511	−10	30	32	212	—	—	—	—	—
Superior Frontal Gyrus	7.0567	−18	4	68	65	—	—	—	—	—
Anterior Cingulate Cortex	—	—	—	—	—	7.3624	8	30	20	25
Supramarginal Gyrus	—	—	—	—	—	4.9477	60	−28	28	23
Inferior Parietal Sulcus	—	—	—	—	—	5.7853	42	−42	34	88
**B) Insula Seed (−43, 14, −4)**										
PositiveImprov > PositiveChromatic										
Region	*Left Hemisphere*					*Right Hemisphere*				
	t-score	x	y	z	Cluster Size	t-score	x	y	z	Cluster Size
Superior Frontal Gyrus	−5.7837	−16	26	52	24	—	—	—	—	—
Middle Frontal Gyrus	−6.7562	−42	22	32	221	—	—	—	—	—
*Middle Frontal Gyrus	—	—	—	—	—	−6.8154	20	0	36	32
*Frontal Eye Fields	—	—	—	—	—	−5.3609	30	2	36	21
Precentral Gyrus	−6.1561	−42	−4	30	89	—	—	—	—	—
Postcentral Gyrus	−6.5393	−36	−34	44	285	—	—	—	—	—
Supramarginal Gyrus	−5.0949	−58	−46	32	20	—	—	—	—	—
Middle Occipital Gyrus	—	—	—	—	—	5.5648	26	−88	14	55
*White matter	—	—	—	—	—	−5.0431	24	44	12	25
NegativeImprov > NegativeChromatic										
	*Left Hemisphere*					*Right Hemisphere*				
	t-score	x	y	z	Cluster Size	t-score	x	y	z	Cluster Size
Superior Medial Gyrus	—	—	—	—	—	6.0208	6	60	18	30
Middle Frontal Gyrus	—	—	—	—	—	−8.0061	32	26	22	90
Inferior Parietal Lobule	−4.8395	−46	−32	36	72	—	—	—	—	—
*Callosum	−8.8971	−20	−20	44	1178	—	—	—	—	—
PositiveImprov > NegativeImprov										
	*Left Hemisphere*					*Right Hemisphere*				
	t-score	x	y	z	Cluster Size	t-score	x	y	z	Cluster Size
Rolandic Operculum	—	—	—	—	—	5.1469	50	−8	12	53
*Substantia Nigra	—	—	—	—	—	−7.6075	6	−12	−14	29
* Cluster was not specified in the Anatomy Toolbox, and was identified using NeuroSynth.								p < 0.01, k > = 20		

All coordinates are presented in the Montreal Neurological Institute template space. Anatomical labels were identified using the SPM Anatomy Toolbox. Results were obtained through generalized psycho-physiological interaction (gPPI) analysis (minimum voxel extent of 20 voxels and p < 0.001 significance threshold, uncorrected).
